# Editorial: Ocular Pharmacology: Recent Breakthroughs and Unmet Needs

**DOI:** 10.3389/fphar.2022.848332

**Published:** 2022-02-14

**Authors:** Claudio Bucolo, Mario Damiano Toro, Chiara M. Eandi

**Affiliations:** ^1^ Department of Biomedical and Biotechnological Sciences, School of Medicine, University of Catania, Catania, Italy; ^2^ Chair and Department of General and Pediatric Ophthalmology, Medical University of Lublin, Lublin, Poland; ^3^ Eye Clinic, Public Health Department, University of Naples “Federico II”, Naples, Italy; ^4^ Department of Ophthalmology, University of Lausanne, Fondation Asile des Aveugles, Jules Gonin Eye Hospital, Lausanne, Switzerland; ^5^ Department of Surgical Sciences, University of Torino, Torino, Italy

**Keywords:** ocular pharmacology, diabetic retinopathy, glaucoma, age-related macular degeneration (AMD), retinoblastoma, cornea

The research on ocular pharmacology has increased significantly over the past decade. More recently, the discovery and approval of eye disease modifying treatments is considered an unbelievable scientific breakthrough. This Research Topic “Ocular Pharmacology: Recent Breakthroughs and Unmet Needs” presents nine *original research articles*, two *reviews*, one *opinion*, and one *brief research report* from eight different Countries, and has contributions that span the field of ocular pharmacology providing new insights on recent drug breakthroughs and medical unmet needs. Most of the contributions concern the retinal conditions that have an important impact on quality-of-life and Countries health systems. Age-related macular degeneration, diabetic retinopathy and glaucoma are the diseases discussed in this Research Topic along with corneal conditions.

Four contributions are related to diabetic retinopathy and diabetic macular edema. The review by Adornetto et al. focused their work on diabetic retinopathy and autophagy, the major catabolic pathway involved in removing and recycling damaged macromolecules, suggesting that dysfunctions of this pathway contribute to the onset and progression of diabetic retinopathy and at the same time could be a good target for therapeutic approach. The authors underlined that most evidence suggests that autophagy may act with a damage/time-dependent double action: under mild stress or during the initial phase of diabetic retinopathy, autophagy acts as an adaptative response with pro-survival and anti-apoptotic effects, whereas, under severe stress and in the later phase of diabetic retinopathy, dysregulated autophagy, because of the system overload due to the prolonged damage, contributes to the apoptotic retinal cell death exacerbating the damage. The high glucose impact has been studied also by Maugeri et al. who used human retinal pigment epithelial cells, to assess the role of p38 MAPK signaling pathway and its modulation by dimethyl fumarate. They demonstrated that dimethyl fumarate treatment attenuated HG-induced apoptosis and protect retinal tissue. The authors concluded that the small molecule dimethyl fumarate represents a good candidate for diabetic retinopathy treatment and warrants further *in vivo* and clinical evaluation. Another important contribution on diabetic retinopathy has been provided by Bucolo et al. who investigated the role of vascular endothelial growth factor (VEGF) at different phases of diabetic retinopathy using *Ins2* ^*Akita*^ (diabetic) mice at different ages. These authors showed that the retina of the diabetic *Ins2* ^*Akita*^ mice, as expected for mice, does not develop proliferative retinopathy even after 46 weeks. However, diabetic *Ins2* ^*Akita*^ mice recapitulate the same evolution of patients with diabetic retinopathy in terms of both retinal neurodegeneration and pro-angiogenic shift, this latter indicated by the progressive protein expression of the pro-angiogenic isoform VEGF-A_164_, which can be sustained by the protein kinase C betaII (PKCβII)/human antigen R (HuR) pathway acting at post-transcriptional level. In agreement with this last concept, this rise in VEGF-A_164_ protein is not paralleled by an increment of the corresponding transcript. Nevertheless, the observed increase in hypoxia inducible factor-1α (HIF-1α) at 9 weeks indicates that this transcription factor may favor, in the early phase of the disease, the transcription of other isoforms, possibly neuroprotective, in the attempt to counteract the neurodegenerative effects of VEGF-A_164_. The authors concluded that the time-dependent VEGF-A_164_ expression in the retina of diabetic *Ins2* ^*Akita*^ mice suggests that pharmacological intervention in diabetic retinopathy might be chosen, among other reasons, based on the specific stages of the pathology to pursue the best clinical outcome. On this regards, innovative ocular drug delivery system such as nanosytems, should be considered to address unmet medical needs (Amadio et al., 2016). Finally, the COVID-19 pandemic situation imposed huge changes in the management of retinal diseases such as diabetic macular edema with a great impact on visual outcome and cost for the patient and the public health system. Iovino et al. proposed their opinion on a possible management paradigm for the treatment of diabetic macular edema in the COVID-19 Era.

Three contributions, two *original research articles* and one *brief research report*, focused on cornea. Roszkowska et al. investigated the effect of oral supplementation with amino acids on corneal nerves regrowth after excimer laser refractive surgery with photorefractive keratectomy (PRK). These authors reported that oral supplementation with eleven amino acids improved significantly corneal nerve restoration after PRK and could thus be considered as an additional treatment during corneal surgical procedures. In an original research study, Du et al. described the corneal protective effects of a water-soluble and biocompatible small molecule, dihydropyrimidinthione derivative, against UV-B damage. Finally, *brief research report* by Bonzano et al. deals with an appropriate method, optical coherence tomography, to monitor the wound healing elicited by cenegermin, a novel recombinant human nerve growth factor, to handle neurotrophic keratitis, a rare orphan condition that affects fewer than 65,000 persons in the United States.

The study from Yousef et al. investigate the safety and efficacy of intravitreal melphalan chemotherapy as a treatment for recurrent and refractory vitreous seeds in patients with retinoblastoma. They concluded that melphalan chemotherapy is an effective and relatively safe treatment modality for retinoblastomas and has changed the outcome of eyes with vitreous seeds, significantly improving the ocular oncologists’ capability to save eyes. However, there are side effects on both the anterior and posterior segments of the eye, and unexpected serious adverse reaction may occur with the standard dose of melphalan.

Two contributions deal with retinal protection and glaucoma. The first is a review leaded by Jassim et al. at University of Pennsylvania regarding the crosstalk between dysfunctional mitochondria and inflammation in glaucomatous neurodegeneration. The review discussed evidence for the interaction between mitochondrial damage and inflammation, with a focus on glaucomatous neurodegeneration, and proposed that positive feedback resulting from this crosstalk drives pathology. The authors underlined that damaged mitochondrial DNA is a damage-associated molecular pattern, which activates the nucleotide-binding domain (NOD)-like receptor protein 3 (NLRP3) inflammasome. They hypothesize that crosstalk between damaged mitochondria and increased inflammatory signaling enhances pathology in glaucomatous neurodegeneration, with implications for other complex age-dependent neurodegenerations like Alzheimer’s and Parkinson’s disease. The second contribution on retinal protection and glaucoma is an original research article on brimonidine, an α_2A_-adrenergic receptor agonist approved for lowering intraocular pressure (IOP) in patients with open-angle glaucoma. The authors investigated the neuroprotective effect of the drug after retinal ischemia damage on mouse eye. They concluded that brimonidine was effective in preventing loss of function of retinal ganglion cells and in regulating inflammatory biomarkers elicited by retinal ischemia/reperfusion injury.

The retrospective study by Fasler et al. confirmed the lack of efficacy of eplerenone, an aldosterone antagonist, versus observation on resolution of subretinal fluid in patients with acute and chronic central serous chorioretinopathy in the real life. These findings are in accordance with previous report (Lotery et al., 2020). Finally, an original research contribution on brolucizumab, the new approved drug for the treatment of neovascular age-related macular degeneration, was published on the present Research Topic. The clinical pharmacological profile and safety has been demonstrated by the HAWK and HARRIER trials (Dugel et al., 2021). However, a few reports so far describe its efficacy in a routine clinical practice. Montesel et al. reported short term real-life experiences of brolucizumab intravitreal injection in a single referral center and showed very good outcomes in fluid control with similar inflammatory side effects as reported in the HAWK and HARRIER trials.
